# Knowledge, attitudes and practices of infection prevention and control among healthcare workers during the COVID 19 pandemic: a descriptive cross-sectional study in three Nigerian states

**DOI:** 10.1186/s12913-023-09218-9

**Published:** 2023-03-14

**Authors:** Bright Orji, Elizabeth Oliveras, Bartholomew Odio, Charity Anoke, Herbert Onuoha, Emmanuel Ugwa, Madeleine Howard, Ibrahim Idris, Edima Akpan, Festus Okoh, Chinyere Nwani, Oniyire Adetiloye, Nwankwo Lawrence, Chioma Oduenyi, Emmanuel Ogharu, Joseph Enne, Folayan W. Abolaji, Rosemary S. Adegbulu, Emily Bryce

**Affiliations:** 1 Jhpiego - an Affiliate of Johns Hopkins University, Abuja, Nigeria; 2 Jhpiego - an Affiliate of Johns Hopkins University, Baltimore, MD USA; 3 Federal Medical Center, Brinin Kudu, Nigeria; 4 Johns Hopkins University School of Public Health, Baltimore, MD USA; 5 State Ministry of Health, Minna, Niger State Nigeria; 6 Reproductive Health Division, Federal Ministry of Health, Abuja, Nigeria; 7 National Malaria Elimination Program, Federal Ministry of Health, Abuja, Nigeria; 8 State Ministry of Health, Abakaliki, Ebonyi State Nigeria; 9 State Ministry of Health, Akure, Ondo State Nigeria

**Keywords:** COVID -19, Pandemic, Knowledge, Attitude, Practice, Infection prevention and control

## Abstract

**Background:**

Emergence of the COVID-19 pandemic created unexpected challenges for health care workers. The global and national supply chain system was disrupted, and affected infection, prevention and control (IPC) practices. This study aimed at documenting health workers knowledge, attitudes and practices (KAP) on IPC in Nigeria during the COVID-19 pandemic.

**Methods:**

The descriptive, mixed-methods cross-sectional study was conducted in Ebonyi, Ondo and Niger states in October 2020. A structured questionnaire was administered to the health workers, complemented by semi-structured interviews that were audio recorded, transcribed and analyzed in Atlas.ti. Quantitative data were entered into REDCap and cleaned, transformed and analyzed using descriptive statistics in SPSS version 25.0 Findings from the qualitative interviews were used to explain the trends observed from quantitative study.

**Results:**

There were demographic differences between community and facility-based health workers in our population. A greater proportion of facility-based providers reported having IPC training compared to community-based health workers ever (p < 0.01) and during the pandemic (p < 0.05). Health care workers had moderate knowledge of general IPC, and attitudes toward and practice of IPC during COVID-19 pandemic. However, the knowledge of the relative effectiveness of prevention measures was low. The mean knowledge scores were greater among facility-based workers compared to community based healthcare workers (p = 0.001). Self-reported IPC practices increased during the pandemic compared to prior to the pandemic, with the exception of the use of N-95 masks and hand sanitizer.

**Conclusion:**

This study found moderate IPC knowledge, attitudes and practices in our study population during the pandemic as compared to pre-pandemic for the study found gaps in correct hand hygienevaried application of different IPC practices to ensure adherence to COVID-19 preventive measures. The study recommends sustained training for IPC and encourages policy makers that budget line specific to COVID-19 response across all the levels of health care delivery will enhance compliance and emergency readiness.

**Supplementary Information:**

The online version contains supplementary material available at 10.1186/s12913-023-09218-9.

**What is already known on this topic**

Proper infection prevention and control is critical at preventing the spread of COVID-19 among health care workers, but disrupted health systems and limited resources restricted facility’s ability to respond adequately.

**What this study adds**

IPC knowledge and practices were reported to be better during the pandemic compared to the pre-pandemic era, but there was variation by cadre of health worker and IPC method.

**How this study might affect research, practice or policy**

This study indicates that cadre-specific training would be beneficial and that when investing in training, government and policy decision makers should invest in resources as well, including PPE supplies.

## Background

The index case of COVID-19 in Nigeria was on February 27th, 2020 and there have since been three major waves, the first lasting until November 30, 2020 during which there were 68,000 cases and 1,173 deaths [1]. The COVID-19 pandemic has disrupted service delivery worldwide, placing an incredible burden on health care workers to provide quality care during a rapidly evolving situation. healthcare workers, including community health workers (CHWs), are at high risk for COVID-19 infection and related mortality [2, 3].

Traditionally, infection prevention and control (IPC) practices are considered the cornerstone for the prevention of infectious diseases [3]. However, COVID-19 overwhelmed health systems with fear, denial, and stigma. Global and national supply chain management systems were disrupted, and IPC practices and compliance among healthcare workers worsened due to inadequate emergency response strategies and the lack of personal protective and hand washing equipment, gloves and sanitizers [4]. Reported lack of training on best IPC practices in the face of COVID-19 raises the chances of contamination [5].

Globally, a number of studies have been conducted to examine HCWs knowledge, attitudes and practices (KAP) of infection prevention and control during COVID-19 period. The results have been varied; many reported correct KAP, but others revealed poor KAP by healthcare workers [5–7]. Multiple studies demonstrated that although HCWs were found to have good knowledge and positive attitudes, this did not necessarily result in good COVID-19 practices of precautionary measures [8–11]. Few studies have specifically assessed community health workers’ KAP towards COVID − 19 in other countries. Previous studies in Nigeria have largely reported positive COVID-19 KAP among HCWs, though these have focused on facility-based providers [12–14].

This study aimed to understand the KAP of facility and community-based health care providers regarding IPC during the first wave of the COVID-19 pandemic in Nigeria. Specifically, we examined (i) if HCWs and CHWs in Nigeria have correct knowledge of IPC guidelines during the COVID-19 pandemic, (ii) if HCWs and CHWs in Nigeria have attitudes about COVID-19 that may hinder service delivery and (iii) if HCWs and CHWs in Nigeria self-report changes in the appropriate use of IPC practices during the COVID-19 pandemic in Nigeria. The Nigeria Federal Ministry of Health, through the National Malaria Elimination Programme (NMEP), requested these data which will be used to help them improve future program planning and implementation while ensuring continuity of essential services during the pandemic. The results of the study are intended to support of the mandate of the Nigeria public health authority.

## Methodology

### Study Design

The descriptive, mixed-methods cross-sectional study was conducted in Ebonyi, Ondo and Niger states in October 2020 at the end of the first wave of COVID-19.

### Study area

The study was conducted in three local government areas (LGAs), Ohaukwu, Akure-South, Bosso, across the three Nigerian states of Ebonyi, Ondo and Niger during October 2020. These LGAs were selected because the parent project, Transforming Intermittent Preventive Treatment for Optimal Pregnancy (TIPTOP) project, was implementing here. The project has been described elsewhere [15]. These states are located in south-east, south-west and north central region respectively and they reflect both Christian and Islamic areas of the country Ohaukwu is a rural LGA with a population of 294,179 (2020) with farming as predominate occupation. Bosso is also rural with a population of 252,076 (2020) with 85% predominantly farmers and 15% on vocational jobs, Whereas Akure-south is an urban though with some rural communities. The LGA has a population of 519,710 (2020) whose occupation includes trading, and civil service (91.4%) and farming (8.6%). There are predominantly public primary health facilities in all three LGAs. There are 63 health facilities (54 public primary health care centers in Ohaukwu, 12 private health care centers and one secondary hospital). Akure South has 50 primary health care centers (4 private, 46 public), and in addition one secondary hospital. While in Bosso there are 68 primary health care centers ( 3 private, 65 public).

### Study Population

All primary level public health centres in the three study LGAs were included. A communal clash in the Effium zone led to closure of all facilities including 8 of the 12 private health facilities in Ohaukwu, Ebonyi State. It was agreed that the remaining 4 would not be a good representative of the private sector practices and therefore the sample was limited to public facilities across all the states. Survey respondents were facility and community health workers on duty at the time that the interview team was present at the facility. Staff were eligible if they had been in their posts for 12 months or more.

### Sampling size

All public health facilities in the three districts of Ohaukwu (Ebonyi State), Akure-south (Ondo state) and Bosso (Niger State) where the TIPTOP project is being implemented were included in the study. In each facility, two facility-based service providers and two CHWs were selected to participate, which resulted in a total sample size of 622 total providers. At the time of study design, estimates of COVID-related knowledge and practice among health care workers in Nigeria were unknown. Therefore, we used an estimate of 50% prevalence, which is widely used when the prevalence is unknown because it represents the largest estimate of variability and produces the most conservative sample size. Using the 50% prevalence estimate and the number of providers surveyed at all project facilities, the margin of error calculated was equal to 5.56%, slightly higher than the standard 5%. The study also included key informant interviews with five facility-based and five community-based providers, five LGA health administrators and three SMoH a total of eighteen interviewees per state. This resulted in total number of fifty-four participants interviewed in the three study LGAs.

### Data source

Quantitative data were collected using an adapted questionnaire that was developed from a previously published questionnaire [8], which includes 10 knowledge questions, 4 questions on attitude and 5 questions on practices pre and during the pandemic. The questionnaire also collected data on demographic characteristics (gender, age, position, professional training, length of time at the facility and number of years of experience) and COVID-19 awareness. A semi structured interview guide was used to collect qualitative information from managers and CHWs affiliated with the recruited health facilities (Annex II). The guide included questions about their current position, perceptions about what worked or did not work in terms of systems and programs put in place to combat COVID-19 at facility, district and LGA levels, and about the availability of infection prevention equipment and commodities. The interviews were audio recorded with permission of the respondents.

Training on the use of qualitative and quantitative tools was conducted for data collectors and supervisors for two days and was immediately followed by one-day of field testing in LGAs not included in the study. Findings from the pretest was used to finalize the study tools.

### Data Analysis

Quantitative data were summarized using descriptive statistics in Stata version 14.2. Knowledge of IPC was assessed using 10 questions and the overall knowledge score reflects the number of questions answered correctly. Attitudes were measured on a 5-point Likert scale and categorized as agree, neutral, or disagree for ease of reporting. IPC practice questions were binary “yes” or “no” responses regarding five different practices pre and during the pandemic. T-tests tests were used to assess whether mean knowledge differed by cadre, and Chi-square and Fisher exact tests were used to assess whether attitudes between community and facility workers and practices pre and during the pandemic were significantly different.

Coding and analysis of qualitative data was carried out by two independent reviewers who worked under a supervising data analyst. Atlas.ti was used, three key themes established prior to the review of the transcripts (PPE use, client contact, resources & supplies); and findings were reported in sections. Though data looked at individual KAP of IPC equipment (hand-gloves, sanitizers, facemasks, etc.) and transmission of malaria and COVID 19, findings were aggregated across health facilities to ascertain how the facility-level health service delivery was affected during the COVID-19 pandemic. Discrepancies in data coding, reporting and findings were resolved among the 2 reviewers in a meeting with the supervising data analyst and the principal investigator. Through this arrangement the authors ensured consistency, and where necessary expanded notes to provide clarity and better understanding. Findings from the qualitative interviews were used to explain the trends observed from quantitative study.

### Ethical approval/consent

Ethical approval was received from the Institutional Review Board of the John Hopkins School of Public Health (JHSPH) as public health surveillance number No: 13,887 and the National Health Research Ethics Committee of Nigeria (Approval Number NHREC/01/01/2007-06/10/2020). Survey data were collected and stored in a secure online application with relational database features housed in a fully customized Jhpiego cloud Server. No personal identifying information was collected from any of the survey or interview participants.

## Results

**Fig. 1:**
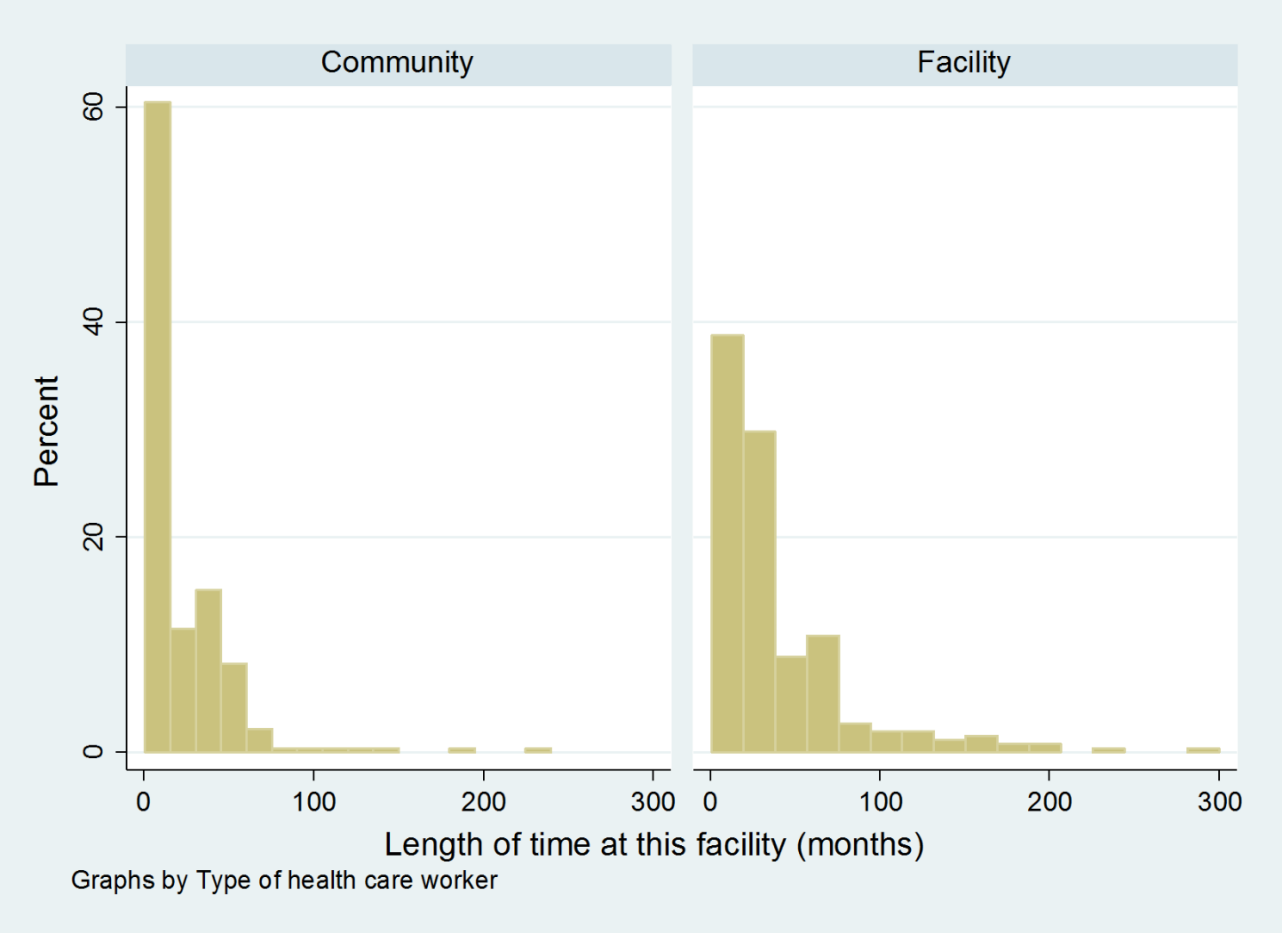
Length of time at facility (months) by provider type

A total of 622 health workers were interviewed and 536 were included in the analysis; providers were excluded if they were missing socio-demographic characteristics or information on knowledge or practice of IPC. The final sample included 278 (52%) CHWs and 258 (48%) facility providers (Table 1). As shown in Fig. 1, a greater proportion of CHWs had been at their post for less than a year, as compared to facility-based providers. Moreover, facility providers were more likely to have been trained on IPC (71.3% vs. 52.9%, p < 0.001). In terms of geographic coverage, slightly more than one third of workers were from Niger state, but the distribution was not significantly different between CHWs and facility providers.

**Table 1:** Characteristics of providers, comparing CHWs and facility-based service providers

	CHWs, n = 278		Facility-based, n = 258		p-value
Parameters	Number	Percentage	Number	Percentage	
**Age**					< 0.001
20s	76	27.3	60	23.3	
30s	107	38.5	62	24.0	
40s	65	23.4	101	39.2	
50+	30	10.8	35	13.6	
**Sex**					< 0.001
Male	88	31.7	40	15.4	
Female	190	68.3	218	84.6	
**Location**					0.563
Ebonyi	89	32.0	88	34.1	
Niger	107	38.5	89	34.5	
Ondo	82	29.5	81	31.4	
**Mean duration at facility (Months)**	24.4 (21.4–27.5)		39.5 (34.2–44.8)		< 0.001
**Attended IPC training (ever)**					< 0.001
No	131	47.1	74	28.7	
Yes	147	52.9	184	71.3	
**Attended IPC training during COVID**					0.045
No	107	38.5	78	30.2	
Yes	171	61.5	180	69.8	

**Fig. 2:**
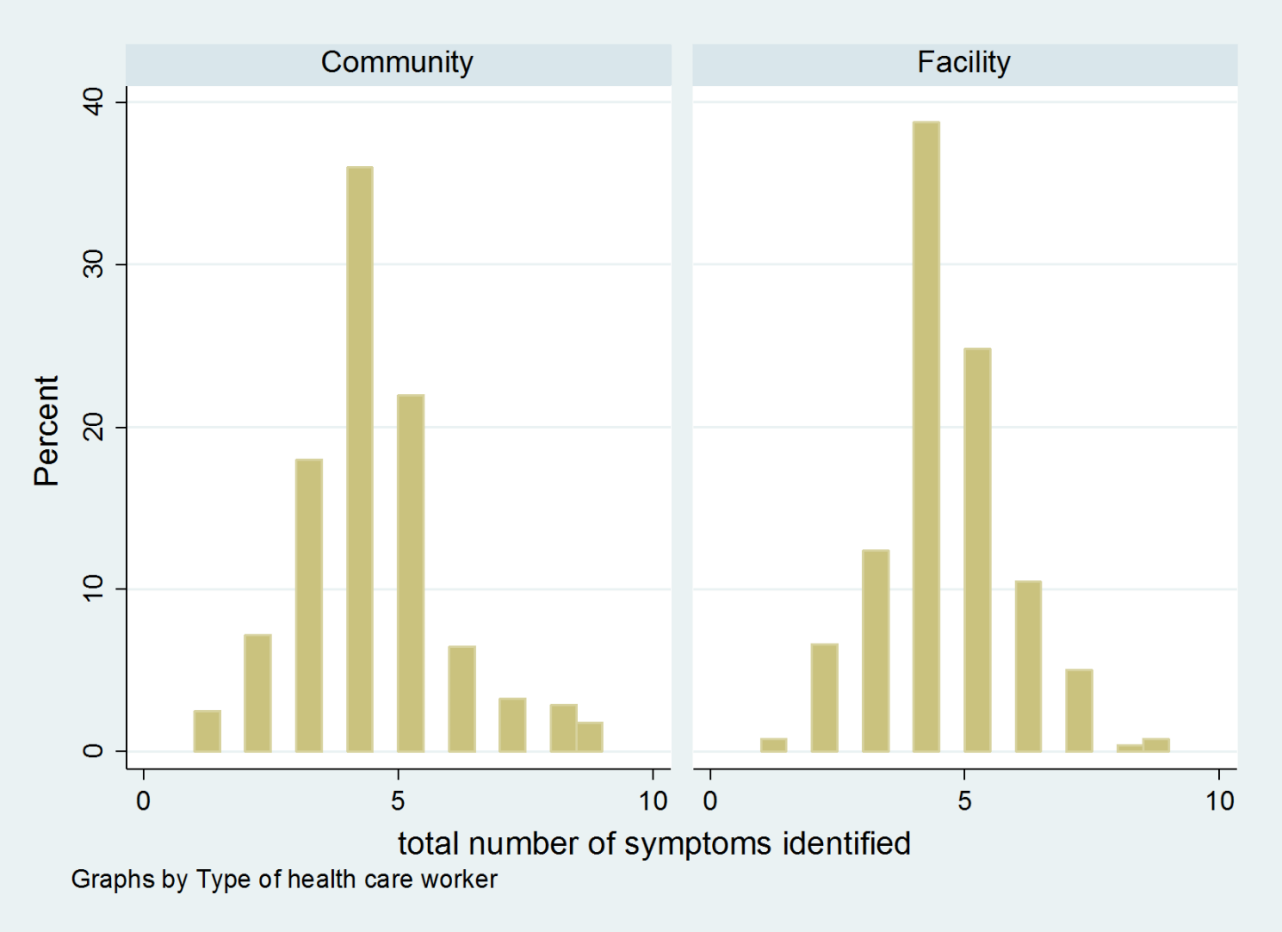
Distribution of the number of COVID-19 symptoms identified (Max = 9)

On the correct knowledge of IPC only 25.6% of providers answered eight or more questions correctly out of the possible ten questions. The overall mean knowledge score for the health workers was 6.46 (95% CI: 6.33–6.59). Facility-based providers scored higher on average (6.69, 95% CI: 6.51–6.87) compared to CHWs (6.26, 95% CI: 6.08–6.44) and the difference was statistically significant (p = 0.001). At the level of individual questions, the difference between cadres was significant for some questions, but not for all. (Table 2). On average, providers named 4.05 (95% CI: 3.93–4.18) possible COVID-19 symptoms out of the nine possible symptoms; there was no difference between CHWs (µ_1_ = 4.0, 95% CI: 3.81–4.19) and facility-based providers (µ_1_ = 4.1, 95% CI: 3.95–4.27) (Fig. 2).

**Table 2:** Number and percent of CHWs and facility providers with correct knowledge about IPC

Questions	CHWs (278) (% correct)	Facility-based (258) (% correct)	p-value
Dirty needles can transmit the disease causing agent	2.5	5.0	0.12
Dirty needles and sharps transmit malaria plasmodium spp	46.4	58.5	0.005
Hand hygiene is an effective method in preventing infection during this COVID-19 pandemic	99.3	98.1	0.27
Use of sterile gloves is the most effective method to prevent infection during this COVID-19 pandemic	29.1	34.5	0.18
Wearing gloves cannot eliminate the need to wash hands	74.5	81.4	0.054
Washing hands before and after examining clients attending ANC is effective in preventing infection transmission	96.8	98.8	0.14
Wearing a face shield does not eliminate the need to use facemasks	62.6	71.7	0.03
The use of facemasks is the most effective method to prevent infection during the COVID-19 pandemic	74.8	70.5	0.27
Hand sanitizers cannot be used effectively when hands are visibly soiled	61.5	64.0	0.56
Use of gowns and aprons is effective in preventing infection transmission during physical examination of a client	78.4	86.4	0.02

Almost three quarters of the surveyed HCWs agreed that social distancing (72%) and lockdown (72%) are unrealistic response to COVID19 pandemic in Nigeria (Table 3). Approximately 90% of HCWs felt that caring for people with COVID 19 could put their families and friends at risk and 94% believed that health care facilities could be a source of infection in the absence of standard precautions. As with knowledge, more facility-based workers agreed with each of the statements than did CHWs, but the difference was only significant with regard to the risks to family and friends (86.0% of CHWs vs. 92.6% of facility workers agreed, p = 0.008).

**Table 3:** Percent of providers who agreed, disagreed, or were neutral about statements about IPC during COVID 19

Statement	Agree	Neutral	Disagree
When caring for a person with COVID-19, you need to worry about putting your family and friends at risk of contracting the disease	89.5	1.5	9.0
In the absence of standard precautions, health care facilities can be the source of infection and epidemic diseases	93.8	2.8	3.4
Social distancing is unrealistic in Nigeria	71.2	3.6	24.2
Total lockdown is an unrealistic response in developing countries like Nigeria	72.0	4.1	23.9

More health workers reported that they practiced recommended IPC behaviors during than before the COVID-19 pandemic (Table 4). The difference was significant with regard to hand washing between patients (66.8% vs. 95.7%), and using a surgical mask in the workplace (61.7% vs. 93.3%). There was no change in reported use of an N95 mask in the workplace (6.5% vs. 7.6%). There was reported incorrect use of hand sanitizer when hands are visibly soiled increased (35.6% vs. 53.2%). Few differences were seen in reported behaviors by type of healthcare worker during the pandemic, although CHWs were more likely to report washing and disinfecting hands after contact with each patient while providing service to clients than were facility providers (98.2% vs. 93.1%, p = 0.003).

**Table 4:** Percent of workers reporting practicing key IPC practices before and during COVID-19

	Percent of workers reporting the behaviour		
	Before COVID	During COVID	p-value for difference
Wash and disinfect hands after contact with each patient when providing ANC services	68.7	95.7	< 0.001
Use hand sanitizer when your hands are visibly soiled	35.5	53.0	< 0.001
Wash your hands in between patients	66.8	95.7	< 0.001
Use a surgical mask in the workplace	52.8	93.3	< 0.001
Use an altered mask (N95) in the workplace	6.3	7.7	0.48

The qualitative interviews supported the quantitative data, showing that IPC practices improved during COVID-19 and elaborated on the ways in which this happened. Key informants in multiple states reported that use of PPEs had increased, and some attributed it directly to the fear of contracting COVID-19:

*We became extremely careful the way we attend to our patients so that we or our clients will not contact COVID-19… We increased our use of face mask, sanitizer, hand glove (Ebonyi)*

*Before, we were conscious that we have to wash our hands. Then we use hand sanitizers too. But face mask, when except may we want to do a procedure. But during the pandemic, everybody is on facemask. (Ondo)*

In addition to increasing use of PPEs, key informants described how COVID-19 resulted in changes in practice in order to limit contact with clients:

*We reduced the number of pregnant women we attend to. We reduced the daily ANC attendance to about 35 by giving longer appointment dates, as against 50–80 which we previously attended to (Ondo)*

The increase in use of PPEs would likely not have been possible without the additional supplies as some key informants noted. For example,

*Supplies of commodities have improved during the pandemic as the LGA and State authorities have ensured that supplies to keep the facility running optimally were provided (KII Ondo)*

*We received hand sanitizers, face masks, three days ago, we received infra-red thermometer. We were also given face shield and apron. We received nose masks, sanitizers and hand gloves. We received infra-red thermometer, hand sanitizers, veronica bucket, liquid soap, hand gloves and face mask (KII, HCW, Ebonyi)*

This was complemented, at least as reported in Ondo state, by technical advice on use of PPE:

*There was an upsurge in the supplies of IPC during pandemic. Let me quickly say again that we have to give kudos to NMA in Ondo State, because the NMA in conjunction with Ministry of Health have an IPC team also moving from one LGA to another and providing the technical know-how on IPC [PPE]. The IPC supplies increase during pandemic as against before the pandemics. (Director PH, Ondo)*

One of the respondents noted their own role in this process:

*…….In this facility, my typical role in fighting COVID-19 is number one. My major role is to create awareness for the people in the community and those that come to the health facility about COVID-19; how it is being spread; how they can prevent it; and how they can go in their day to day activities still protecting themselves from the infection and prompt treatment of anyone that comes to the clinic (KII, Ondo)*

## Discussion

This study assessed COVID-19 related KAP of community and facility-based healthcare workers in three LGAs in Nigeria. Both cadres of health workers had moderate IPC knowledge, with only a quarter answering 80% or more of the questions correctly, and it was found that they had poorer knowledge about the relative effectiveness of different methods for preventing transmission of COVID-19. Furthermore facility-based providers performed better than CHWs in mean knowledge. Despite the lower knowledge, providers reported increased IPC practices during the pandemic as compared to before in both the surveys and key informant interviews. This was attributed to increased availability and use of PPE, bolstered by government support and technical assistance. However, the reported use of a N95 mask and hand sanitizer remained low. Finally, although the HCWs believe that their work at the facility puts themselves and their families at risk, they do not believe that societal actions (lockdown or social distancing) are realistic in Nigeria.

The knowledge scores and proportion scoring 80% or better in our study is much lower compared to previous studies in Nigeria and other countries [5, 6, 13, 14, 16]. This may be in part due to the fact that only two-thirds of respondents reported receiving training on IPC during the pandemic in our study population. However, it is important to note that our questionnaire asked about relative effectiveness (i.e., facemasks are the most effective measure) versus other study’s questionnaires that ask about absolute effectiveness (i.e., facemasks are an effective measure) or about PPE generally (using PPE is a way to prevent COVID-19). This could have resulted in misinterpretation of the question amongst our participants, whereby they responded to an absolute instead of relative effectiveness question, which led to the lower scores. Additionally, the first question “Can dirty needles transmit disease-causing agent?” had few correct responses, particularly compared to other questions in the survey. Again, potentially there could have been confusion around “causes COVID-19” versus “causes any disease”, resulting in the very low proportion of correct responses.

The lower knowledge scores among CHWs compared to facility-based providers has been reported in other studies as well [8, 14, 17]. In our population, this association is likely explained by the fact that a lower proportion of CHWs in our study that received IPC training during the pandemic compared to facility-based providers. Additionally, the demographic differences reported between the two cadres of health worker may have contributed to the differences in knowledge as well, including age and time at post.

The result from Table 4 indicated that majority of the health workers self-reported changes in the use of IPC during the pandemic compared to pre-pandemic era. The improvement was greater for use of surgical mask, hand sanitizer, gloves and hand washing. Improved COVID-19 prevention practices have been shown in other studies as well, but unlike our study, in these studies there are also high overall knowledge scores [5, 17, 18]. Interestingly, Kamacooko et al. reported high overall knowledge, but poor COVID-19 prevention practices [8]. As mentioned earlier, the low reported knowledge in our study may have been due to question misinterpretation, particularly given the improved IPC practices by both cadres of providers. Additionally, the good preventative practices were most likely influenced by the PPE availability reported by participants, which is not the case in other studies [2, 17].

The vast majority of respondents indicated that they worry about putting family and friends at risk of contracting the disease, believed that health facilities could be a source of infection and followed good COVID-19 prevention practices while at work. Fear of contracting COVID-19 or spreading it to family members is commonly cited in other studies [2, 17, 18]. The fear may have increased adherence to preventive practices, as posited by another author [14]. Ejeh et al. reported that although knowledge and preventative practise in the facilities was high, they reported low use of facemasks leaving the house [13]. Similarly in our population, despite high prevention adherence, with the exception of N95 masks, more than a quarter of respondents believed that societal actions to prevent COVID-19 spread were not realistic in Nigeria.

A limitation of this study is the potential bias from self-report for the key IPC practices prior to and during the pandemic. Social desirability bias could have inflated the proportion of respondents who reported key IPC practices during the pandemic. As mentioned earlier, a second limitation is the potential confusion over the question wording, which resulted in lower knowledge scores.

## Conclusion

This study found moderate IPC knowledge and COVID-19 prevention practices, fuelled by adequate PPE supplies and fear of spreading the virus. Based on these findings, the government should increase training for and the capacity of various health workers as well as sensitization on adherence and preventive measures to reduce infection rates. Given the lower reported training amongst CHW, the government should invest in cadre-specific training and resources to ensure all cadres are properly trained in IPC for COVID-19. Furthermore, different learning and management approaches, like hands-on training or supportive supervision could be beneficial in improving practice along with knowledge, though it’s important to acknowledge the difficult of providing this sort of support during a pandemic, particularly in the early stages. Finally, policy makers should include a budget line specific to COVID-19 response across all the levels of health care delivery in order to enhance compliance and emergency readiness.

## Electronic supplementary material

Below is the link to the electronic supplementary material.

Supplementary Material 1

Supplementary Material 2

## Data Availability

The datasets, including de-identified provider quantitative and qualitative data, used and/or analyzed during the current study are available from the corresponding author on reasonable request.
