# Supplementary material for: Knowledge, attitudes and practices of infection prevention and control among healthcare workers during the COVID 19 pandemic: a descriptive cross-sectional study in three Nigerian states
Source: BMC Health Serv Res. 2023 Mar 14;23:253. doi: 10.1186/s12913-023-09218-9 (PMC10013228; doi:10.1186/s12913-023-09218-9)
Supplement: Supplementary file 1 — Supplementary Material 1 [file 12913_2023_9218_MOESM1_ESM.docx]

**Health Care Workers Assessment**

Knowledge, attitudes, and practices of frontline health care providers (including CHWs) on infection prevention and control before and during the COVID-19 pandemic in Nigeria

| **INTERVIEW INFO** |  |
| --- | --- |
|  | 1. Date of Interview:   --------(date)/---------( month)/--------(year) |
| 1. LGA/State: | 3. Start Time: ___ ___ : ___ ___  4. End Time: ___ ___ : ___ ___ |
| 5. Interviewer' s Name: | 6. Recorder's Name: |
| **RESPONDENT INFO** |  |
| 7. Age (decade; *circle one*): 20s 30s 40s 50s | 8. Gender (*Circle one*): Male Female |
| 9. Position at this facility: | 10. Professional Training (*Circle one*):  1. Medical doctor  2. Nurse  3. Midwife  4. Nurse/Midwife  5. CHEWs |
| 11. Length of Time in this facility: | 12. Number of Years of Work Experience: |

INTRODUCTION

*Introduce yourself and your role*

*Explain: National Malaria Elimination Program (NMEP)* is collaborating with the TIPTOP project and other stakeholders to assess the knowledge, attitude and practices of health workers (including community health workers) on infection prevention and control during the COVID-19 pandemic. Findings from the assessment will inform recommendations on strategies to improve and sustain service delivery at the facility level during and after the COVID-19 pandemic.

*Obtain written consent from participants.*

Thank you for speaking with me today. We would like to hear your thoughts and experience of IPC before and during the COVID-19 pandemic through this assessment. Please remember your responses are confidential.

**Health Care Worker Awareness of COVID-19**

| **Statement** | **Response(Circle one)** |  |
| --- | --- | --- |
| 1. Have you heard anything about the Coronavirus Covid-19? | 1 Yes  2 No | If no, stop the interview |
| 1. Do you think COVID-19 is an acute problem in society? | 1 Yes  2 No |  |
| 1. Have you encountered a person infected by COVID-19 while working? | 1 Yes  2 No |  |
| 1. Have you admitted, cared for, or generally managed a patient suspected of having COVID-19? | 1 Yes  2 No |  |
| 1. Are you afraid of getting COVID-19 while performing your duties in the health facilities? | 1 Yes  2 No |  |
| 1. Do you think people are afraid of transferring the disease from work to family? | 1 Yes  2 No |  |
| 1. How is COVID-19 disease transmitted? Please write down as many options as mentioned. | 1 _______________  2 _______________   1. ________________ 2. ________________ |  |
| 1. What are the symptoms of a COVID-19 disease?   *Please circle all that apply* | 1 Fever  2 Dry Cough  3. Tiredness  4. Aches and pains  4. Diarrhea  5. Loss of taste or smell  6. Difficulty in breathing/shortness of breath  7. Chest pain/pressure  8. Loss of speech or movement  9. Other  Others specify_______ |  |
| 1. Does COVID-19 have more severe symptoms in people with underlying diseases? | 1 Yes  2 No | [skip to Q23] |
| 1. If yes, please mention those diseases that you know can make coronavirus symptoms more severe?   *Circle all mentioned diseases* | 1. Diabetes  2. High blood pressure  3. Cancer  4. Immune-suppressive diseases (HIV, tuberculosis etc.) |  |
| 1. Is malaria related to COVID-19 in any way? | 1 Yes  2 No | [skip to Q25] |
| 1. If yes, what are the similarities | 1 Fever  2 Dry Cough  3. Tiredness  4. Aches and pains  4. Diarrhea  5. Loss of taste or smell  6. Difficulty breathing/shortness of breath  7. Chest pain/pressure  8. Loss of speech or movement |  |
| 1. Can people with mild symptoms transmit the disease to other people during the incubation period? | 1 Yes  2 No |  |
| 1. Are people with severe symptoms the only ones who can transmit the disease to others? | 1 Yes  2 No |  |
| 1. Is taking antibiotics effective in preventing or treating the COVID-19? | 1 Yes  2 No |  |
| 1. Is taking Chloroquine/Hydroxychloroquine effective in preventing or treating the COVID-19? | 1 Yes  2 No |  |

**Health Care Workers’ Knowledge about infection prevention and control**

| **Statement** | **Response (circle one)** |  |
| --- | --- | --- |
| 1. Have you ever attended training on Infection prevention and control (IPC)? | 1 Yes  2 No | If no, skip to Q.31 |
| 1. If yes, was this in the last two years? | 1 Yes  2 No |  |
| 1. What of training on Infection Prevention and Control (IPC) in the midst COVID-19 pandemic? | 1 Yes  2 No |  |
| 1. Can dirty needles transmit the COVID 19 -causing virus? | 1 Yes  2 No |  |
| 1. Can dirty needles and sharps transmit malaria plasmodium spp? | 1 Yes  2 No |  |
| 1. Is hand hygiene an effective method in preventing infection during this COVID-19 pandemic? | 1 Yes  2 No |  |
| 1. Is the use of sterile gloves the most effective method to prevent infection during this COVID-19 pandemic? | 1 Yes  2 No |  |
| 1. Can wearing gloves eliminate the need to wash hands? | 1 Yes  2 No |  |
| 1. Is washing of hands before and after examining clients attending ANC effective in preventing infection transmission? | 1 Yes  2 No |  |
| 1. Do wearing a face shield eliminate the need to use facemasks? | 1 Yes  2 No |  |
| 1. Is the use of facemasks the most effective method to prevent infection during the COVID-19 pandemic? | 1 Yes  2 No |  |
| 1. Can hand sanitizers be used effectively when the hand is visibly soiled? | 1 Yes  2 No |  |
| 1. Is the use of gowns and aprons effective in preventing infection transmission during physical examination of a client? | 1 Yes  2 No |  |

**Health care workers’ (HCWs) attitude toward infection prevention and control**

| **Statement** | **Strongly agree** | **Agree** | **Neutral** | **Disagree** | **Strongly disagree** |
| --- | --- | --- | --- | --- | --- |
| 1. When providing services to pregnant women attending ANC, health workers should not carry out physical examination without using hand gloves | **1** | **2** | **3** | **4** | **5** |
| 1. When caring for a person with COVID-19, you need to worry about putting your family and friends at risk of contracting the disease | **1** | **2** | **3** | **4** | **5** |
| 1. Healthcare workers in your facility worry about being infected while caring for clients during this COVID-19 pandemic | **1** | **2** | **3** | **4** | **5** |
| 1. Glove use for all client or patient care contacts is a useful strategy for reducing risk of transmission of organisms | **1** | **2** | **3** | **4** | **5** |
| 1. In the absence of standard precautions, health care facilities can be the source of infection and epidemic diseases | **1** | **2** | **3** | **4** | **5** |
| 1. The risk of occupational infection among health workers in your work place is high | **1** | **2** | **3** | **4** | **5** |
| 1. Government response to the emergence of COVID-19 pandemic was timely | **1** | **2** | **3** | **4** | **5** |
| 1. Response to the pandemic was appropriate | **1** | **2** | **3** | **4** | **5** |
| 1. IPC supplies were more available during the COVID-19 compared to before the pandemic | **1** | **2** | **3** | **4** | **5** |
| 1. Malaria symptoms and signs are similar to COVID-19 | **1** | **2** | **3** | **4** | **5** |
| 1. Global response to COVID-19 has been greatly exaggerated | **1** | **2** | **3** | **4** | **5** |
| 1. COVID-19 is a disease of rich countries | **1** | **2** | **3** | **4** | **5** |
| 1. Africans have greater resistance to COVID-19 compared to Europeans and Americans | **1** | **2** | **3** | **4** | **5** |
| 1. COVID-19 affects mostly older people with underlying health conditions | **1** | **2** | **3** | **4** | **5** |
| 1. Most Nigerians do not take the COVID-19 pandemic seriously | **1** | **2** | **3** | **4** | **5** |
| Wearing of facemasks is the single most important intervention to stop community spread of COVID-19 | **1** | **2** | **3** | **4** | **5** |
| Social distancing is unrealistic in Nigeria | **1** | **2** | **3** | **4** | **5** |
| Total lockdown is an unrealistic response in developing countries like Nigeria | **1** | **2** | **3** | **4** | **5** |

**Health care workers’ (HCWs) practices toward COVID-19**

| **Statement** | **Response (Circle one)** |  |
| --- | --- | --- |
| 1. When providing ANC services, did you wash and disinfect your hands after contact with each patient before COVID-19 Pandemic started? | 1 Yes  2 No |  |
| 1. When providing ANC services, do you wash and disinfect your hands after contact with each patient amidst the COVID-19 pandemic i.e. during the pandemic? | 1 Yes  2 No |  |
| 1. At the ANC clinic, did you use hand sanitizer when your hands were visibly soiled before the COVID-19 pandemic started? | 1 Yes  2 No |  |
| 1. At the ANC clinic, do you use hand sanitizer when your hands are visibly soiled during the COVID-19 pandemic? |  |  |
| 1. When providing ANC services, did you wash your hands before glove use before the -COVID-19 pandemic? | 1 Yes  2 No |  |
| 1. When providing ANC services, do you wash your hands after glove use in the midst of the COVID-19 pandemic? | 1 Yes  2 No | **If No, skip to Q.66** |
| 1. If yes, please describe the steps you take in washing your hands when providing ANC services? Please circle all steps mentioned by the respondents | 1. Wet hands with water  2. Apply enough soap to cover all hand surfaces  3. Rub hands palm to palm  4. Right palm over left dorsum with interlaced fingers and vice versa  5. Palm to palm with fingers interlaced  6. Backs of fingers to opposing palms with fingers interlocked  7. Rotational rubbing of left thumb clasped in right palm and vice versa  8. Rotational rubbing, backwards and forwards with clasped fingers of right hand in left palm and vice versa  9. Rinse hands with water  10. Dry hands thoroughly with a single use towel  11. Use towel to turn off faucet  12. Your hands are now safe |  |
| 1. Did you wash your hands in between patients before the pandemic? | 1 Yes  2 No |  |
| 1. Do you wash your hands in between patients before and amidst the pandemic? | 1 Yes  2 No |  |
| 1. Did you know how to wear facemasks the right way before the COVID-19 pandemic? | 1 Yes  2 No |  |
| 1. Do you know how to wear facemask the right way amidst the COVID-19 pandemic? | 1 Yes  2 No | **If no, skip to Q.72** |
| 1. If yes, can you explain to us the right way to wear a facemask? Please circle al steps mentioned by the respondents | 1.Wash your hands before touching the mask  2. Inspect the mask for tears or holes  3. Find the top-side where the metal piece or stiff edge is  4.Ensure the colored-side faces outwards  5. Place the metal piece or stiff edge over your nose  6. Cover your mouth, nose, and chin  7. Adjust the mask to your face without leaving gaps on the sides  8. Avoid touching the mask  9. Remove the mask from behind the ears or head  10. keep the mask away from you and surfaces while removing it  11. Discard the mask immediately after use preferably into a closed bin  12. Wash your hands after discarding the mask |  |
| 1. Did you use a surgical mask in the workplace before the pandemic? | 1 Yes  2 No |  |
| 1. Do you use a surgical mask in the workplace during the pandemic? | 1. Yes  2. No |  |
| 1. Did you use an altered mask (N95) in the workplace before the COVID-19? | 1 Yes  2 No |  |
| 1. Do you use an altered mask (N95) in the workplace during the COVID-19? | 1 Yes  2 No |  |
| 1. Do you keep the distance (at least 2 meter) during contact with the patient? | 1 Yes  2 No |  |
| 1. How do you differentiate malaria from COVID-19?   *Please write all that the respondent mentioned* |  |  |
| 1. With which patients do you adhere to the above principles? | 1 No patients  2 Patients with severe COVID symptoms  3 Patients with mild COVID symptoms  4 All patients |  |

**Availability of IPC supplies in health facilities during the COVID-19 pandemic**

| **Review stock card/bin cards to assess whether there has been a stock out of IPC supplies in the last three months** | **Yes** | **No** |
| --- | --- | --- |
| Sterile hand gloves |  |  |
| Hand sanitizer |  |  |
| Surgical masks |  |  |
| Facemasks (N95) |  |  |
| Face shields |  |  |
| Gowns |  |  |
| Aprons |  |  |
| Chlorine solution |  |  |
| Tap water |  |  |
| Well water |  |  |
| Harvested Rain water |  |  |
| Tanker water supply |  |  |

Thank you for your time!
