# Supplementary material for: Knowledge, attitudes and practices of infection prevention and control among healthcare workers during the COVID 19 pandemic: a descriptive cross-sectional study in three Nigerian states
Source: BMC Health Serv Res. 2023 Mar 14;23:253. doi: 10.1186/s12913-023-09218-9 (PMC10013228; doi:10.1186/s12913-023-09218-9)
Supplement: Supplementary file 2 — Supplementary Material 2 [file 12913_2023_9218_MOESM2_ESM.docx]

**Key Informant Interview Guide for Facility Managers and SMOH/LGA**

**Documenting knowledge, attitudes, and practices of frontline health care providers (including CHWs) on infection prevention and control during COVID-19 pandemic in Nigeria**

| **INTERVIEW INFO** |  |
| --- | --- |
|  | Date of Interview:  --------(date)/---------( month)/--------(year) |
| Region: | Start Time: ___ ___ : ___ ___  End Time: ___ ___ : ___ ___ |
| Interviewer' s Name: | Recorder's Name: |
| **RESPONDENT INFO** |  |
| Age (decade; circle one): 20s 30s 40s 50s | Gender (Circle one): Male Female |
| Position: | Number of Years of Work Experience: |

INTRODUCTION

*Introduce yourself and your role*

*Explain: National Malaria Elimination Program (NMEP)* is collaborating with the TIPTOP project and other stakeholders to assess the knowledge, attitude and practices of health workers (including community health workers) on infection prevention and control during the COVID-19 pandemic. Findings from the assessment will inform recommendations on strategies to improve and sustain service delivery at the facility level amidst and after the COVID-19 pandemic.

*Obtain written consent from the participants.*

Thank you for speaking with me today. We would like to hear your thoughts and experience of Infection Prevention and Control before and during the COVID-19 pandemic through this assessment. Please remember your responses are confidential.

- Current position and role in improving health outcomes at this facility/LGA/State
  - Can you start by telling me about your current position and role?
    - Can you tell me about your role in fighting COVID-19 pandemic in your facility?
    - What are the systems put in place by your facility/LGA/State to fight COVID-19?
    - How does providing malaria services differ during the COVID-19 pandemic compared to before the pandemic?
    - How did health workers differentiate malaria from COVID-19?
- Impressions of and experience with malaria service delivery during the COVID-19 pandemic
  - What malaria services are being provided in the COVID-19 pandemic period?
  - Can you tell me the challenges faced by health workers providing those services?
  - What has worked in terms of systems/programs put in place to combat COVID-19 at the facility/district/region?
  - Does the facility/district/facility have a rapid response team for COVID-19?
- Availability of infection prevention equipment
  - How were IPC supplies before and during the pandemic?
  - How did the pandemic affect IPC supplies?
  - What the infection prevention equipment was provided for health workers to fight the COVID-19 pandemic? Were these used?
  - Please can you tell me which ones were actually used, or not used?
  - Do health workers have the skill required for the use of this equipment? *Probe for details.*
  - What, if anything, was done to ensure proper and appropriate use of the equipment?
  - How did the availability or non-availability of IPC equipment affect service delivery during the pandemic?
- Availability of commodity at the health facility
  - How did the COVID-19 pandemic affect the availability of commodities in the health facility?
  - How did the availability or non-availability of commodities affect service delivery in the facility during the pandemic?
